# Multivalent Inactivated Vaccine Protects Chickens from Distinct Clades of Highly Pathogenic Avian Influenza Subtypes H5N1 and H5N8

**DOI:** 10.3390/vaccines13020204

**Published:** 2025-02-19

**Authors:** Walid H. Kilany, Marwa Safwat, Mohamed A. Zain El-Abideen, Islam Hisham, Yasmine Moussa, Ahmed Ali, Magdy F. Elkady

**Affiliations:** 1 Reference Laboratory for Veterinary Quality Control on Poultry Production (RLQP), Animal Health Research Institute (AHRI), Agricultural Research Center (ARC), P.O. Box 264, Dokki, Giza 12618, Egypt; marwasafwat13@yahoo.com (M.S.); zien_vet@yahoo.com (M.A.Z.E.-A.); 2 MEVAC—Middle East for Vaccines, Second Industrial Area, El-Salhya El-Gededa 44813, Egypt; y.mousa@me-vac.com; 3 Poultry Diseases Department, Faculty of Veterinary Medicine, Beni-Suef University, Beni-Suef 62511, Egypt; ahmed.ali1@vet.bsu.edu.eg (A.A.); mfelkady@vet.bsu.edu.eg (M.F.E.)

**Keywords:** avian influenza, highly pathogenic avian influenza virus, chicken, H5N1, H5N8, HPAI, vaccine

## Abstract

Background/Objective: Highly pathogenic avian influenza (HPAI) H5 subtype remains a significant menace to both the poultry industry and human public health. Biosecurity and mass vaccination of susceptible commercial poultry flocks are crucial to reduce the devastating economic loss and hinder the evolution of the virus. Methods: In this study, we developed a multivalent avian influenza virus (AIV) vaccine, including strains representing the HPAI 2.2.1.1., 2.2.1.2., and 2.3.4.4b clades circulating in Egypt and the Middle East. Specific pathogen-free (SPF) two-week-old chickens were vaccinated with a single vaccine shot and observed for four weeks post-vaccination before being challenged. The challenge experiment involved using one strain of HPAI H5N1 subtype clade 2.2.1.2 and two strains of HPAI H5N8 subtype clade 2.3.4.4b derived from chickens and ducks. To assess the vaccine’s potency and efficacy, the pre-challenge humoral immune response and post-challenge survival and virus shedding were evaluated. **Results:** All the vaccinated birds exhibited 100% seroconversion 2 weeks post-vaccination (2 WPV). In addition, protective antibody titers against each diagnostic antigen, i.e., 7.8 ± 1.8 (H5N1, clade 2.2.1.2), 10.0 ± 0.0 (H5N1, clade 2.2.1.1), and 7.5 ± 0.9 (H5N8, clade 2.3.4.4b) were detected 3 WPV. The vaccination achieved complete protection (100%) against all challenge viruses with no disease symptoms. The vaccinated birds exhibited a statistically significant reduction in oropharyngeal virus shedding 2 days post-challenge (DPC). Conclusions: This study illustrated that a single application of a multivalent genetic-matching whole AIV vaccine under laboratory conditions elicits adequate protection against the HPAI challenge, representing 2.2.1.2 and 2.3.4.4b clades. The developed vaccine has the potential to be a vaccine of choice against a broad range of HPAI in commercial flocks raised under field conditions in endemic areas.

## 1. Introduction

The goose/Guangdong (gs/GD) lineage of avian influenza H5 viruses has undergone consistent genetic and antigenic changes, resulting in the formation of several clades since 1996. These clades exhibit different levels of pathogenicity for avian hosts [1]. Avian influenza viruses (AIVs) are named after their main surface proteins, i.e., hemagglutinin (HA) and neuraminidase (NA). Of the 18 HA and 11 NA subtypes identified, 16 HA and 9 NA subtypes have been found in birds [2]. Based on their ability to cause disease in chickens, avian influenza viruses are classified as low pathogenicity (LPAI) or highly pathogenic (HPAI).

HPAI H5N1 used to be the most common subtype of the Gs/GD lineage. However, in 2008, new NA subtypes of Gs/GD lineage HPAIVs belonging to the third order clade 2.3.4 were discovered in China. Later on, the HA underwent further diversification, resulting in the emergence of other subgroups categorized under the fourth order, i.e., clade 2.3.4.4 [3]. The exchange and reassortment of gene segments with LPAI viruses in domestic ducks and wild birds have evolved the viruses under clade 2.3.4.4 H5Nx into eight distinct fifth order HA genetic subgroups (a–h) and numerous genotypes [4]. The ancestral strain of the HPAIV H5N8 subtype was obtained from ducks in China in 2010 and classified as clade 2.3.4.4. Over time, the strains have developed into clade 2.3.4.4a (resembling the Buan/Donglim strain) and 2.3.4.4b, which was initially identified in Korean birds (resembling the Gochang strain) [5].

Wild and domestic waterfowl have served as the natural host and primary reservoir of influenza A viruses [6,7]. Infected ducks often did not show disease symptoms; meanwhile, chickens exposed to HPAI viruses exhibited severe clinical signs and high mortality rates [8]. Widespread infections, impairment of the nervous system, and notable mortality in ducks were linked to HPAI H5N1viruses that emerged in Asia between 2002 and 2005 [9,10]. Consistently, HPAI H5N8 viruses that emerged in Asia have spread from ducks to infect other poultry species, e.g., chickens and turkeys [11,12]. Mallard ducks were linked to the genetic reassortment of HPAI viruses by the evolution of genetically distinct strains [13]. Several variables may contribute to the disease course and the immunological response of domestic chickens and ducks to HPAI viruses [11,14].

HPAI vaccination is a contentious issue in terms of its effectiveness as a means of prevention and control. Some experts have suggested that mismatched vaccination could contribute to an increase in HPAI cases in poultry and possibly contribute to the emergence of a pandemic in humans due to immune pressure [15]. Many, however, view vaccination as a crucial tool for effectively controlling HPAI and providing long-term health benefits to birds and humans [16]. Several AIV vaccines have been developed, including inactivated whole viral vaccines, recombinant virus vector vaccines, subunit vaccines, and DNA vaccines [17,18]. Conventional whole viral vaccines based on naturally occurring or genetically modified LPAI seeds represent the majority of the authorized AIV vaccines globally. To provide protection against a field virus, the vaccine seed should be epidemiologically relevant or closely linked genetically or antigenically [17,19]. Several reports have addressed the added value of multivalent vaccines for offering robust protection against HPAI viruses [20,21,22].

AIV vaccines can be evaluated either by a direct approach, via assessment of clinical protection against clinical signs and mortality, or by reducing the number of challenges of virus shedding through an experimental model [23]. In parallel, the indirect approach for AIV vaccine evaluation includes assessing the induced humoral immune response by serological testing, e.g., HI or ELISA [2]. Antibody titers have a positive predictive value in determining the effectiveness of vaccines in protecting against a challenge from HPAI strains [24]. The World Organization of Animal Health (WOAH) Terrestrial Manual stated that the minimum post-vaccinal serological titers using HI test should be at least 5 log_2_ and 7 log_2_ in order to protect from mortality or decrease the challenge of virus replication and shedding, respectively. Furthermore, a standardized vaccine efficacy evaluation scheme includes at least an 80% survival rate against challenge with a representative HPAI strain able to induce 90% mortality in the sham population [18].

This study aimed to assess the effectiveness of a whole inactivated multivalent virus vaccine against the HPAI viruses representing distinct clades. The vaccine contained AIVs representing clades 2.2.1.1 (H5N1), 2.2.1.2 (H5N1), and 2.3.4.4b (H5N8). The evaluation approach included post-vaccinal serological monitoring, clinical protection, and the reduction in the shedding titers after exposure to challenge viruses derived from different poultry species.

## 2. Materials and Methods

### 2.1. Viruses

Reassortant AIV H5N1 (A/chicken/Eg/RG-173CAL/2017) and (RgA/chicken/Egypt/ME1010/2016) belonging to clades 2.2.1.2 (acc. no. MG192005.1) and 2.2.1.1. (acc. no. MH558951.1), respectively, were developed previously by the Reference Laboratory for Veterinary Quality Control on Poultry Production (RLQP), Animal Health Research Institute (AHRI), Egypt [19]. Reassortant AIV H5N8 (rgA/chicken/ME-2018/H5N8) belonging to clade 2.3.4.4b (acc. no. MW193074.1) was developed by MEVAC—Middle East for Vaccines, Giza, Egypt [20].

HPAI challenge viruses A/chicken/EG/1575S (H5N1 clade 2.2.1.2., EPI_ISL_174424), A/chicken/Egypt/A4/2021 (H5N8 clade 2.3.4.4b, acc. no. ON024718), A/duck/Egypt/FAOSL4/2021 (H5N8 clade 2.3.4.4b, acc. no. ON024724) were provided by RLQP, Giza, Egypt and selected to estimate the vaccine efficacy in the experimental model. All the viruses were propagated and titrated using 10-day-old specific pathogen-free (SPF) embryonated chicken eggs (ECE) obtained from Nile SPF eggs, Kom Oshiem, Fayoum, Egypt. Virus titration was performed by inoculating ten-fold serial dilutions of the virus samples into the allantoic sacs of SPF ECE, as previously described [25].

### 2.2. HA Gene Sequence Analysis

Available sequences of AIV H5Nx isolated from avian species were retrieved from the GISAID Data Science Initiative—EpiFlu™ database (GISAID https://gisaid.org/; Munich, Germany). Sequences were filtered down using the following criteria: Hemagglutinin H5 subtype, reported in Egypt (H5N8 subtype clade 2.3.4.4b, H5N1 subtype clades 2.2.1.1 and 2.2.1.2), and complete segments for HA, and years between 2016 and 2021. This approach resulted in 171 strains (clade 2.3.4.4b), 77 strains (clade 2.2.1.1), and 298 strains (clade 2.2.1.2). The HPAI strains were compared to the vaccine viruses. Deduced amino acid sequences were determined using Geneious^®^ 7.1.3 (Biomatters Ltd., Auckland, New Zealand).

### 2.3. Virus Inactivation and Vaccine Formulation

The virus harvests were inactivated using 0.2% formalin (*v*/*v*) (Merck KGaA, Darmstadt, Germany). The hemagglutination activity of the inactivated virus harvests was calculated using the hemagglutination test [18]. Inactivation completion was evaluated by inoculating three successive passages into 10-day-old SPF-ECE via the allantoic route. The antigen bulks were then mixed with MEVAC™ IMA 70 adjuvant (MEVAC, El-Salhya El-Gededa, Egypt) composed of liquid paraffin, Polysorbate 80, and Sorbitan oleate. The antigen aqueous phase was pre-emulsified with the oil adjuvant at a 30:70 ratio (*w*/*w*) at 1000 rpm using the Silverson^®^ L5M high-shear laboratory mixer (Silverson Machines, Inc., Buckinghamshire, UK). The speed of the mixer was then increased to 3000 rpm and maintained for 30 min for the emulsification of 10,000 doses of the vaccine. The temperature of the mixture was kept between 18 and 22 °C during the emulsion formulation process. The formulated product was given the name MEFLUVAC™ H5 Plus 8 (MEVAC, El-Salhya El-Gededa, Egypt). Before being employed in animal trials, the produced emulsion was examined for its physicochemical properties, bacterial and fungal sterility [26], and freedom from mycoplasma [27].

### 2.4. Evaluation of the Vaccine’s Physicochemical Properties

The conductivity of the vaccine was measured by JENWAY^®^ Model 4520 conductivity meter (Cole-Parmer^®^, Villepinte, France), while the vaccine’s viscosity was estimated by Brookfield viscometer (AMETEK^®^ Brookfield, PA, USA). The vaccine samples were centrifuged at 6000 rpm for 15 min using a CYAN Tabletop centrifuge (Cypress Diagnostics, Hulshout, Belgium) to determine the emulsion stability. The pH of the vaccine was measured using the JENWAY^®^ Model 3510 Standard Digital pH Meter (Cole-Parmer^®^, Villepinte, France).

### 2.5. Birds and Experimental Design

One hundred five (*n* = 105) SPF chickens, 2 weeks of age (2 WOA) (Nile SPF eggs, Kom Oshiem, Fayoum, Egypt), were allocated to the study. Forty-five birds (Group A, GA) were vaccinated with MEFLUVAC™ H5 Plus 8 vaccine (0.5 mL/bird) via intramuscular injection in the thigh muscles; however, the remaining sixty birds (Group B, GB) were inoculated with an equal volume of normal saline instead. Birds were reared separately in chicken isolators at the BSL-3 Laboratory Animal Facility, Reference Laboratory for Veterinary Quality Control on Poultry Production (RLQP), Animal Health Research Institute (AHRI), Egypt. Feed and water were available ad libitum. Sera were collected from sixteen birds from each group on a weekly basis for 4 weeks post-vaccination (4 WPV).

### 2.6. Measurement of Antibody Response

The hemagglutination inhibition (HI) test was carried out according to the WOAH manual [18] to monitor the post-vaccination humoral immune response using the diagnostic antigens representing AIV H5N1 subtype clades 2.2.1.1, 2.2.1.2, and AIV H5N8 subtype clade 2.3.4.4b viruses. Briefly, twofold serial dilutions of sera were mixed with four hemagglutination units of the diagnostic antigen. The HI titer was determined using a 1% red blood cell (RBC) suspension collected from at least three SPF chickens at least 8 weeks of age. Arithmetic means of HI titers were expressed as reciprocal log_2,_ and inhibition of hemagglutination at a dilution ≥2 log_2_ was considered a specific antibody positive to AIV. The seroconversion rates were estimated as the proportion of birds with positive HI titers and were calculated using the following formula [28]:

(1) $$Seroconversion\;rate\;(\%)=\frac{Number\;of\;positive\;birds\;with\;HI\;titers\;(\geq 3.0\;log2)}{Number\;of\;tested\;birds}\times 100$$

### 2.7. Challenge Experiment

**Figure 1:**
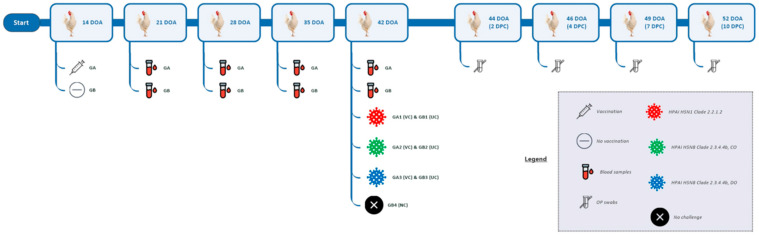
Scheme of the experimental design. DOA: day of age, GA: Group A, GB: Group B, VC: vaccinated challenged, UC: unvaccinated challenged, NC: negative control, CO: chicken origin, DO: duck origin, DPC: days post-challenge.

At 6 weeks of age, the experimental birds of GA and GB were randomly divided into seven groups (*n* = 15 chicks/group) before conducting the challenge experiment. The vaccinated GA1 and the unvaccinated GB1 were challenged with HPAI virus resembling H5N1 subtype clades 2.2.1.2. Similarly, vaccinated GA2 and the unvaccinated GB2 were challenged with HPAI virus H5N8 subtype clade 2.3.4.4b (chicken origin, CO); meanwhile, vaccinated GA3 and the unvaccinated GB3 were challenged with HPAI virus H5N8 subtype clade 2.3.4.4b (duck origin, DO). Each challenged bird received 100 μL containing 6.0 log_10_ EID_50_ from the particular HPAI viruses via the oculonasal route using a calibrated micropipette. Birds allotted to GB4 were inoculated with an equal volume of normal saline and then kept as a negative control (NC) sham. Chickens were monitored for 10 days post-challenge (10 DPC) for clinical signs and mortality, and the observations were recorded daily until the end of each trial (Table 1 and Figure 1). The mean death time (MDT) was calculated as the average death time at 24 h intervals for each challenge strain utilized in GB1-GB3 [29].

### 2.8. Viral Re-Isolation and Detection

Oropharyngeal (OP) swabs were sampled 2, 4, 7, and 10 DPC. Samples were collected in a 1 mL phosphate-buffered saline, pH 7.2, containing gentamycin (50 μg/mL) and nystatin (1000 U/mL) (20). Swab samples were vortexed and then centrifuged at 2000 rpm for 10 min. at 4 °C to pellet the debris. The supernatants of individual samples were tested via virus titration to monitor virus shedding in 10-day-old SPF ECE. The infective dose (EID_50_/mL) was then calculated [30].

### 2.9. Ethics Statement

The challenge experiment was carried out inside BSL-3 chicken isolators at the Reference Laboratory for Veterinary Quality Control on Poultry Production (RLQP), Animal Health Research Institute (AHRI), Agricultural Research Center (ARC), Egypt. The study was designed to minimize animal suffering and was conducted in conformity with the provisions related to ARRIVE Guidelines 2.0 [31]. All the experimental procedures were reviewed and approved by the Animal Care and Use Committee (ACUC) (no. RLQP-VP20-2107).

### 2.10. Statistical Analysis

The variations in titer shedding between groups were analyzed using one-way ANOVA with Tukey’s post-test, as appropriate, using GraphPad Prism version 7.00 for Windows (GraphPad Software, San Diego, CA, USA, www.graphpad.com). A *p*-value < 0.05 was considered statistically significant. Descriptive statistics was used to describe the results of the physicochemical properties of the investigated product as well as the antibody titers of the tested vaccine groups using Microsoft^®^ Excel software, Microsoft^®^ Corporation, Redmond, WA, USA. A Kaplan–Meir curve was used to illustrate the cumulative survival of the experimental groups during the observation period.

## 3. Results

### 3.1. Sequence Analysis of the AIV Strains

In general, the vaccine strains demonstrated a high amino acid percentage of identity, with their HPAI strains representing their counterpart clades. The reassortant LPAI H5N8 subtype vaccine strain, belonging to clade 2.3.4.4b, had an amino acid sequence identity range of 97–100% with the HPAI H5N8 subtype clade 2.3.4.4b strains isolated from avian species in Egypt. The reassortant LPAI H5N1 subtype clade 2.2.1.2 and 2.2.1.1 vaccine strains exhibited an identity range of 94.6–98.6% and 93.2–96.3% with their analogous clades, respectively (Table 2).

The sequenced HA proteins of the HPAI challenge viruses in this investigation included multiple basic amino acids at their cleavage sites (KRRKKR/GLF for the HPAI H5N1 subtype and KRRK-R/GLF for the H5N8 subtype). Nevertheless, the reassortant vaccine strains exhibited a monobasic cleavage site, indicating the LPAI criteria. The amino acid residues H103, E186, G221, Q222, and G224 (H5 numbering) of all the studied strains indicated specificity towards α-2,3-linked sialic acid receptors present in the intestinal and respiratory tracts of birds (Table 3).

Chicken-originated HPAI H5N8 subtype clade 2.3.4.4b had the amino acid S129 linked to epitope A (eA), whereas duck-originated HPAI and the LPAI H5N8 strains of the same clade had the amino acid L129. However, a deletion in site 129 was consistently noticed in the examined AIV H5N1 clade 2.2.1.2 strains. Q115L and D124N associated with antigenic site (AS) and epitope A (eA) were observed in the AIV H5N8 subtype strains compared to H5N1 clade 2.2.1.2. Epitope B (eB) V174I, P181S, and R189N sites were consistently noticed in AIV H5N8 strains. Amino acids anticipated to correlate with epitope C (eC) were identical across all examined strains. Reassortant LPAI H5N1 subtype clade 2.2.1.1 vaccine strain showed T151I, A185E, and Q192K representing epitope D (eD) compared to the rest of the investigated strains. Amino acids predicted to be associated with epitope E (eE) exhibited complete identity among all the AIV H5N8 strains. Amino acid substitutions N43D, L71I, N72R, K82R, and I83A were observed in AIV H5N8 clade 2.3.4.4b strains compared to H5N1 clade 2.2.1.2; meanwhile, AIV H5N1 clade 2.2.1.1 strain demonstrated 71P and 83T (Table 3).

The receptor binding sites (RBSs) of the HA protein showed N94S in all the AIV H5N8 strains. All the RBSs exhibited complete identity between AIV H5N1 subtype 2.2.1.2 clade studied strains. AIV H5N8 subtype strains of different clades possessed S133A, D183N, and S223R. N-glycosylation sites were consistently detected. The analysis of the established sequences revealed the presence of six N-linked glycosylation sites at positions 11, 23, 165, 286, 483, and 542 (Table 3).

### 3.2. Vaccine’s Physicochemical Properties

No critical deviations from the predetermined acceptance limits of the physicochemical properties of the tested final container samples (*n* = 5) of the product were detected. The prepared water-in-oil (W/O) emulsion was proven homogenous and stable. The average conductivity, viscosity, and pH values were 0.001 ± 0.003 µS/cm, 54.4 ± 0.5 mPa.s, and 6.32 ± 0.07, respectively. Also, the inspected samples were proven sterile without any detectable contaminating micro-organisms, i.e., bacteria, fungi, or mycoplasma, under the conditions of the tests.

### 3.3. Post-Vaccination Humoral Immune Response

**Figure 2:**
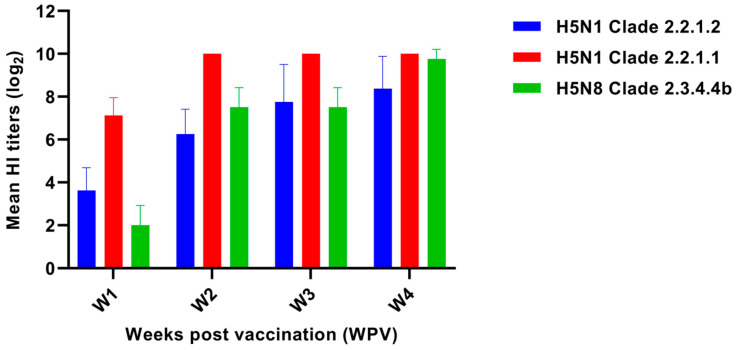
Profile of weekly log_2_ HI antibody responses of chickens vaccinated with MEFULVAC™ H5 Plus 8 against diagnostic antigens resembling AIV H5N1 subtype clades 2.2.1.1, 2.2.1.2, and AIV H5N8 subtype clade 2.3.4.4b. Chickens were vaccinated at 2 weeks of age (2 WOA)**.** Data are presented as the mean ± standard deviation (SD).

Serological responses against diagnostic antigens representing AIV H5N1 subtype clades 2.2.1.1 and 2.2.1.2 and H5N8 subtype clade 2.3.4.4b were detected in all the vaccinated birds (Figure 2). The H5N1 clade 2.2.1.2 antigen elicited an average log_2_ titer of 3.6 ± 1.1 log_2_ HIU 1 WPV, with a seroconversion rate of 88% in the vaccinated GA. The average immune response significantly increased to 6.3 ± 1.2 log_2_ HIU, and all birds (100%) exhibited seroconversion 2 WPV. The trend persisted at 3 and 4 WPV, with average immune responses of 7.8 ± 1.8 and 8.4 ± 1.5 log_2_ HIU, respectively, and a seroconversion rate of 100% (Table 4).

All the GA vaccinated birds (100%) showed seroconversion for the H5N1 clade 2.2.1.1 antigen 1 WPV with an average immune response of 7.1 ± 0.8 log_2_ HIU. The high response observed in the following weeks remained consistent, with a mean immune response of 10.0 ± 0.0 log_2_ HIU at 2, 3, and 4 WPV, indicating complete seroconversion (Table 4).

The H5N8 clade 2.3.4.4b antigen elicited a mean immune response of 2.0 ± 0.9 log_2_ HIU at 1 WPV, with only 13% of the vaccinated birds of GA demonstrating seroconversion. In contrast, the mean immune response markedly increased to 7.5 ± 0.9 log_2_ HIU 2 WPV, with a 100% seroconversion rate. The immune response was sustained at 3 and 4 WPV, with mean HI titer values of 7.5 ± 0.9 and 9.8 ± 0.5 log_2_ HIU, respectively, and complete seroconversion rates. The unvaccinated control GB lacked any detectable HI titers, denoting the study’s validity (Table 4).

### 3.4. Challenge Experiment in SPF Chickens

**Figure 3:**
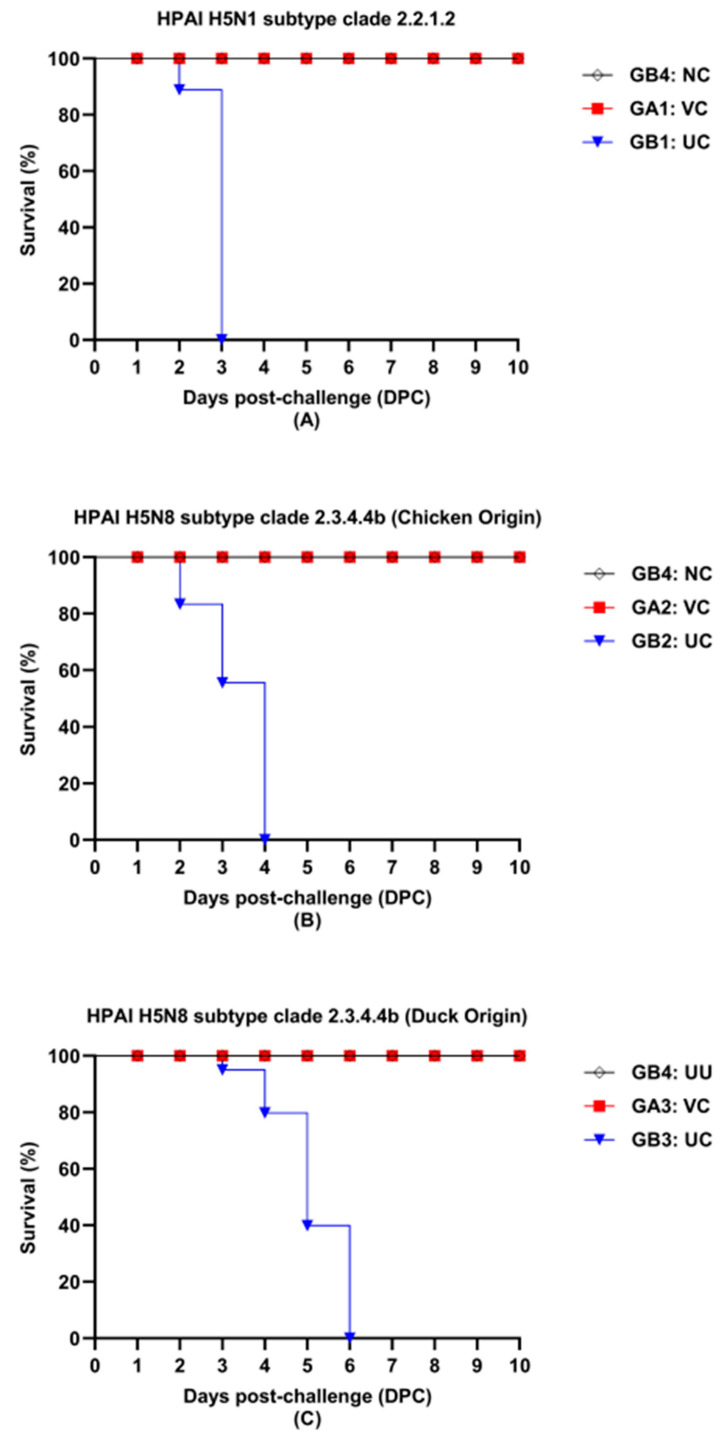
Survival curves of unvaccinated and vaccinated SPF chickens after challenge with 0.1 mL of 10^6.0^ EID_50_ of HPAI H5N1 subtype clade 2.2.1.2 (**A**), HPAI H5N8 subtype clade 2.3.4.4b virus (chicken origin) (**B**), and HPAI H5N8 subtype clade 2.3.4.4b virus (duck origin) (**C**). NC: negative control, VC: vaccinated challenged, UC: unvaccinated challenged. Chickens were challenged at 6 weeks of age (6 WOA).

The vaccinated challenged (VC) chickens of GA1-GA3 were clinically protected against the challenge viruses, while the unvaccinated challenged (UC) GB1-GB3 developed obvious clinical signs by 2–3 DPC. Clinical signs comprised ruffled feathers, lethargy, and dyspnea. Only two to three birds in GA1-GA3 displayed ruffled feathers after challenge; however, all the birds recovered 5DPC. The unvaccinated sentinels in GB4 showed no detectable signs of disease (Supplementary Table S1). All the vaccinated birds survived the challenges; meanwhile, the challenged controls showed 100% mortality. Mortality percentage in unvaccinated GB1 challenged with HPAI H5N1 subtype clade 2.2.1.2 virus was 100% 3 DPC with an MDT of 2.8 (Figure 3A). Unvaccinated GB2 challenged with HPAI H5N8 subtype clade 2.3.4.4b virus (chicken origin) demonstrated 100% mortality 4 DPC with an MDT of 3.0 (Figure 3B). Interestingly, all the unvaccinated birds in GB3 challenged with HPAI H5N8 subtype clade 2.3.4.4b virus (duck origin) died at 6 DPC with an MDT of 4.6 (Figure 3C).

All the VC GA1-GA3 excreted significantly low levels of HPAI viruses in OP swabs after the challenge with at least a −2.0 log_10_ difference compared to the analogous UC control GB1-GB3 (Table 5). Two DPC, six out of fifteen birds (40%) tested positive in GA1 challenged with HPAI H5N1 subtype clade 2.2.1.2. The mean value decreased to 2.5 ± 0.36 log_10_ EID_50_, with only 3 out of 15 birds (20%) testing positive 4 DPC. The mean value of shedding in GA2 challenged with HPAI H5N8 subtype clade 2.3.4.4b (chicken origin) was 3.5 ± 0.26 log_10_ EID_50_, and 5 out of 15 birds (33%) tested positive 2 DPC. The shedding titers dropped ~1.0 log_10_ EID_50_, with 3 out of 15 birds (20%) testing positive 4 DPC. The lowest shedding titers and numbers of shedders were observed in GA3 challenged with HPAI H5N8 subtype clade 2.3.4.4b (duck origin). Four out of 15 birds (27%) tested positive 2 DPC, then decreased to 2.4 ± 0.0 log_10_ EID_50_ with 2 out of 15 positive birds (13%) 4DPC. At 7 DPC and 10 DPC, all birds (100%) of the VC GA1-GA3 tested negative for shedding viruses. Nonetheless, OP sampling of GB1-GB3 was unfeasible as all the challenged birds had succumbed 4–6 DPC (Figure 3). All the NC ‘sham’ birds (100%) in GB4 tested negative for virus detection at all the sampling points (Table 5).

## 4. Discussion

The HPAI viruses inflict significant economic harm on the poultry industry and raise public health concerns due to their potential for zoonotic transmission [6,33]. HPAI H5N1 viruses of clade 2.2.1.1 have been reported in Egypt since 2008; meanwhile, strains belonging to clade 2.2.1.2 were identified in 2012 [34]. Since 2014, HPAI H5N8 clade 2.3.4.4 emerged in South Korea and has affected a wide range of wild birds and commercial poultry in Asia, America, Africa, and Europe [35]. The virus was reported in Egypt for the first time in late 2016 in migratory birds during an active surveillance program [36]. The virus subsequently disseminated to terrestrial poultry populations, leading to enormous mortalities [37,38,39]. Although *Galliformes* (landfowl) and *Anseriformes* (waterfowl) are commonly afflicted by AIVs, even with diversified clinical patterns, the development, dissemination, and persistence of the HPAI H5N8 subtype have been associated with both domestic and wild aquatic birds [5]. The variation in pathogenicity between the two species can be ascribed to changes in their immune responses and susceptibility to the virus [40].

The primary strategies for curbing the spread of HPAIVs are stamping out, biosecurity measures, and immunization. In China, integration between the three strategies was considered crucial for controlling the spread of AIVs effectively [41,42]. In Egypt, vaccination of susceptible commercial terrestrial poultry was prioritized due to the significant allocation of resources required for culling diseased birds or controlling live bird markets. Nevertheless, a national vaccination strategy was not executed in a manner comparable to the Chinese model [43]. Multiple reports have determined the beneficial impact of genetically matched AIV vaccines [19,20].

Comprehending the specific molecular factors that cause the gradual changes in the antigenic properties of HA glycoprotein is essential for developing vaccines against H5 avian influenza viruses [44,45]. To provide comprehensive defense against AIVs, genetically matched vaccines should stimulate the production of antibodies that specifically target conserved viral epitopes, such as the RBS [24,46]. Multivalent vaccines utilized in endemic regions have demonstrated immunogenicity and robust protection against circulating HPAI viruses [47,48].

In this study, a trivalent inactivated reassortant of whole AIV antigens representing three distinct clades, 2.2.1.1, 2.2.1.2, and 2.3.4.4b, combined with an oil adjuvant, was developed. The vaccine was designed to confer protection against a broad range of HPAI strains circulating in Egypt and the Middle East. An experimental model based on single-dose vaccine administration to SPF chickens was used to evaluate the vaccine. The vaccinated birds exhibited complete seroconversion as well as protective antibody levels, i.e., ≥5 log_2_ against each of the vaccine fractions were seen two weeks after vaccine application. Although the H5N8 vaccine fraction had the lowest seroconversion rate in the first week following vaccination, no interference was noticed in the subsequent weeks. These results are consistent with prior findings from the bivalent form of the vaccine [20]. The peak levels of antibodies were seen four weeks after immunization. The vaccine met the potency criteria outlined in the WOAH Terrestrial Manual [18].

Clinical signs, mortality, and oropharyngeal virus shedding are widely used to estimate the effectiveness of the vaccine against challenges [43,49]. HPAI H5-subtype challenge viruses representing clades 2.2.1.2 (H5N1) and 2.3.4.4b (H5N8) derived from chicken and duck origins were used for efficacy assessment of the vaccine. The vaccine induced 100% protection against the challenge viruses used. The pre-existing humoral antibody levels before the experimental challenge iterated the favorable correlation with optimal protection [50,51]. The majority of the vaccinated birds showed no clinical signs associated with the HPAI challenges; meanwhile, the observed mild clinical signs were resolved by 5 DPC.

In contrast, the unvaccinated challenged birds showed severe clinical signs 1 DPC. HPAI H5N8 subtype clade 2.3.4.4b viruses were reported to be associated with lower pathogenicity in poultry species compared to HPAI H5N1 viruses [52]. It is worth noting that despite none of the unvaccinated controls having survived the experimental challenge, each of the HPAI strains resulted in different mortality patterns. The duck-originated HPAI H5N8 subtype challenge strain took six days, resulting in 100% mortality with the longest MDT, i.e., 4.6 days; however, four days were sufficient for the chicken-originated strain with an MDT of 3.0. HPAI H5N1 subtype clade 2.2.1.2 strain achieved 100% mortality by 3 DPC with an MDT of 2.8. Similar findings were attributed to the species from which the HPAI strains were isolated [52,53]. A comparative pathogenicity evaluation of the challenge of HPAI viruses would provide a better understanding of virus pathobiology and transmission.

Vaccines are crucial in providing clinical protection, decreasing the amount of virus shedding, and providing enduring immunity against HPAI viruses [54]. Efficient implementation of an immunization strategy is crucial because of the zoonotic repercussions of HPAI outbreaks, highlighting the necessity for control measures [55]. The vaccinated groups had at least a twofold reduction in the overall number of birds shedding the virus compared to the unvaccinated groups 2 DPC. Consistently, no challenge viruses were found in the vaccinated groups 7 DPC. These findings adhere to the potency levels specified in WOAH. Quantitative PCR (qRT-PCR) is capable of sensitive detection and determining the amount of viral excretion; however, titration of oropharyngeal swabs in SPF ECE provided reliable information on the virus infectivity in the tested samples. Further evaluation of the multivalent vaccine in commercial birds will emphasize its potency and effectiveness under field conditions.

## 5. Conclusions

The developed multivalent vaccine was potent as determined by the post-vaccinal immune response and effective as indicated by clinical protection, survival rates, and reduced oropharyngeal virus shedding against challenge with HPAI H5 subtype representing clades 2.2.1.2 and 2.3.4.4b derived from chicken and duck origins. It is recommended that the combined vaccine be evaluated under field conditions with commercially raised birds, such as broilers or layers/breeders.

## Figures and Tables

**Table 1:** Experimental design of vaccination trial and post-vaccination challenge study.

Group(s) ^i^	Vaccination, 2 WOA		Challenge, 6 WOA	
	Treatment	Dose/Route	Treatment ^ii^	Dose/Route
GA (*n* = 45)	Vaccine	0.5 mL/IM inj.	GA1 (*n* = 15): HPAI H5N1 clade 2.2.1.2 (VC)	100 µL, 6.0 log_10_ EID_50_/Oculonasal
			GA2 (*n* = 15): HPAI H5N8 clade 2.3.4.4b, CO (VC)	
			GA3 (*n* = 15): HPAI H5N8 clade 2.3.4.4b, DO (VC)	
GB (*n* = 60)	Saline	0.5 mL/IM inj.	GB1 (*n* = 15): HPAI H5N1 clade 2.2.1.2 (UC)	
			GB2 (*n* = 15): HPAI H5N8 clade 2.3.4.4b, CO (UC)	
			GB3 (*n* = 15): HPAI H5N8 Clade 2.3.4.4b, DO (UC)	
			GB4 (*n* = 15): NC	100 µL/oculonasal

^i^ Abbreviations: WOA: weeks of age, IM inj.: intramuscular injection, VC: vaccinated challenged, UC, unvaccinated challenged, NC: negative control, CO: chicken origin, DO: duck origin. ^ii^ Experimental GA and GB were divided into equal subgroups (GA1, GA2, GA3, GB1, GB2, GB3, and GB4) at 6 WOA.

**Table 2:** Amino acid identity of MEFLUVAC™ H5 Plus 8 vaccine reassortant seed viruses compared to Egyptian HPAI H5N1 and H5N8 subtypes.

S	Vaccine Strains	Accession No.	HPAI H5N8 Clade 2.3.4.4b (*n* = 171) ^iii^	HPAI H5N1 Clade 2.2.1.2 (*n* = 298)	HPAI H5N1 Clade 2.2.1.1 (*n* = 77)
1	H5N8 2.3.4.4b; rgA/CK ^iv^/ME-2018/H5N8	MW193074.1	97.0–100% ^v^	88.7–91.7%	88.9–91.2%
2	H5N1 2.2.1.2; A/CK/Egypt/RG-173 CAL/2017	MG192005.1	90.3–92.4%	94.6–98.6%	92.1–95.3%
3	H5N1 2.2.1.1; RgA/CK/Egypt/ME1010/2016	MH558951.1	88.9–91.0%	89.4–92.9%	93.2–96.3%

^iii^ Number of retrieved complete HA protein sequences from GISAID EpiFlu™ platform and aligned using MUSCLE. ^iv^ Chicken. ^v^ (min–max).

**Table 3:** Characteristic amino acid substitutions in antigenic site (AS), receptor binding site (RBS), and N-glycosylation site (GS) of the haemagglutinin protein of the vaccine and challenge strains.

Amino Acid Residue No.	HPAI Challenge Strains			Reassortant LPAI Vaccine Strains			Predicted HA Domain or Structure [32]
	EPI_ISL_174424 H5N1 2.2.1.2	ON024718.1 H5N8 2.3.4.4b CO ^vi^	ON024724.1 H5N8 2.3.4.4b DO ^vii^	MW193074.1 H5N8 2.3.4.4b	MG192005.1 H5N1 2.2.1.2	MH558951.1 H5N1 2.2.1.1	
43	N	D	D	D	. ^viii^	D	eE ^ix^
45	D	N	N	N	.	.	-
53	R	K	K	K	.	.	AS
71	L	I	I	I	.	P	eE
72	N	R	R	R	.	.	eE
74	P	.	.	.	.	S	-
82	K	R	R	R	.	R	eE
83	I	A	A	A	.	T	eE
94	N	S	S	S	.	.	-
95	F	L	L	L	.	.	-
115	Q	L	L	L	.	K	AS eA ^x^
120	D	S	S	S	N	S	-
123	S	P	P	P	.	P	-
124	D	N	N	N	.	.	AS eA
127	A	T	T	T	T	.	eA
129	- ^xi^	S	L	L	-	L	RBS/eA
133	S	A	A	A	.	.	-
140	R	A	A	T	G	G	AS/eB ^xii^
141	S	P	P	P	P	P	AS/eB
144	F	.	.	.	.	Y	eB
151	T	I	I	I	.	I	eD ^xiii^
155	D	.	.	.	.	N	eD
156	A	.	.	.	.	T	eD
162	K	I	I	I	.	E	-
165	N	.	.	.	.	H	GS
169	Q	R	R	R	.	.	-
174	V	I	I	I	.	.	eB
181	P	S	S	S	.	.	eB
183	D	N	N	N	.	.	-
184	A	.	.	.	.	E	-
185	A	E	E	E	.	E	AS eD
189	R	N	N	N	.	.	AS/RBS
190	L	.	.	.	.	I	RBS
192	Q	K	K	K	.	K	eD
195	T	.	N	.	.	.	-
218	K	Q	Q	Q	.	.	RBS
223	S	R	R	R	.	.	-
226	M	.	.	.	.	V	eD
227	E	D	D	D	.	.	-
235	S	P	P	P	.	.	-
238	A	.	.	.	.	T	-
240	N	H	H	H	.	.	-
252	N	Y	Y	Y	.	.	-
268	E	G	G	.	.	.	-
269	L	V	V	V	.	.	-
270	E	.	.	.	K	.	-
272	S	G	G	G	.	.	-
273	N	H	H	H	.	.	-
275	N	.	.	.	.	S	-
282	I	V	V	V	.	V	-
310	R	K	K	K	.	.	-

^vi^ Chicken origin. ^vii^ Duck origin. ^viii^ Residues that are identical to CK/Eg/1575S/2015, ISL_174424, H5N1 2.2.1.2. The studied receptor binding and antigenic sites are previously reported by (Duvvuri, et al., 2009 [32]). ^ix^ Epitope E. ^x^ Epitope A. ^xi^ Not found, deletion. ^xii^ Epitope B. ^xiii^ Epitope D.

**Table 4:** Post-vaccination HI mean antibody titers (log_2_) and seroconversion rates in SPF chickens vaccinated with MEFLUVAC™ H5 Plus 8 against AIV H5N1 subtype clades 2.2.1.1 and 2.2.1.2 and H5N8 subtype clade 2.3.4.4b diagnostic antigens.

Group(s) ^xiv^	Antigen	Weeks Post-Vaccination (WPV)							
		1		2		3		4	
		Titer ^xv^	% ^xvi^	Titer	%	Titer	%	Titer	%
GA	AIV H5N1, Clade 2.2.1.2	3.6 ± 1.1	88%	6.3 ± 1.2	100%	7.8 ± 1.8	100%	8.4 ± 1.5	100%
GB		Nd ^xvii^	N/A ^xviii^	Nd	N/A	Nd	N/A	Nd	N/A
GA	AIV H5N1, Clade 2.2.1.1	7.1 ± 0.8	100%	10.0 ± 0.0	100%	10.0 ± 0.0	100%	10.0 ± 0.0	100%
GB		Nd	N/A	Nd	N/A	Nd	N/A	Nd	N/A
GA	AIV H5N8, Clade 2.3.4.4b	2.0 ± 0.9	13%	7.5 ± 0.9	100%	7.5 ± 0.9	100%	9.8 ± 0.5	100%
GB		Nd	N/A	Nd	N/A	Nd	N/A	Nd	N/A

^xiv^ Group A (n = 45): vaccinated, Group B (n = 60): unvaccinated. ^xv^ The mean hemagglutination inhibition titer ± SD is expressed as log_2_ HIU. ^xvi^ $Seroconversion\;rate\;(\%)=\frac{Number\;of\;positive\;birds\;with\;HI\;titers\;(\geq 3.0\;log2)}{Number\;of\;tested\;birds}\times 100.$ ^xvii^ Not detected. ^xviii^ Not applicable.

**Table 5:** Virus shedding titers (log_10_ EID_50_/mL) on the 2nd, 4th, 7th, and 10th day post challenge (DPC).

Group(s) ^xix^ (*n* = 15 for Each Group)	Days Post Challenge (DPC)							
	2		4		7		10	
	Titer ^xx^	Positivity ^xxi^	Titer	Positivity	Titer	Positivity	Titer	Positivity
GA1: VC-2.2.1.2	3.2 ± 0.00 ^b^	6/15 (40%)	2.5 ± 0.36 ^b^	3/15 (20%)	0.0 ± 0.00 ^a^	0/15 (0%)	0.0 ± 0.00 ^a^	0/15 (0%)
GA2: VC-2.3.4.4b, CO	3.5 ± 0.26 ^b^	5/15 (33%)	2.6 ± 0.29 ^b^	3/15 (20%)	0.0 ± 0.00 ^a^	0/15 (0%)	0.0 ± 0.00 ^a^	0/15 (0%)
GA3: VC-2.3.4.4b, DO	2.5 ± 0.30 ^b^	4/15 (27%)	2.4 ± 0.00 ^b^	2/15 (13%)	0.0 ± 0.00 ^a^	0/15 (0%)	0.0 ± 0.00 ^a^	0/15 (0%)
GB1: UC-2.2.1.2	5.6 ± 0.25 ^c^	12/12 (100%)	N/A ^xxii^	N/A	N/A	N/A	N/A	N/A
GB2: UC-2.3.4.4b, CO	5.5 ± 0.21 ^c^	10/10 (100%)	N/A	N/A	N/A	N/A	N/A	N/A
GB3: UC-2.3.4.4b, DO	4.5 ± 0.25 ^c^	15/15 (100%)	4.0 ± 0.27 ^c^	9/9 (100%)	N/A	N/A	N/A	N/A
GB4: NC	0.0 ± 0.00 ^a^	0/15 (0%)	0.0 ± 0.00 ^a^	0/15 (0%)	0.0 ± 0.00 ^a^	0/15 (0%)	0.0 ± 0.00 ^a^	0/15 (0%)

^xix^ VC: vaccinated challenged, UC: unvaccinated challenged, NC: negative control, CO: chicken origin, DO: duck origin. ^xx^ The mean shedding titer ± SD is expressed as log_10_ EID_50_/mL. Virus titers in the same column followed by different small letters are statistically different (*p* ˂ 0.05). ^xxi^ Positivity %: percentage of active virus shedder birds out of the total number of the tested samples. ^xxii^ Not applicable.

## Data Availability

The data presented in this study are available in this article and the Supplementary Materials.

## Supplementary Materials

The following supporting information can be downloaded at: https://www.mdpi.com/article/10.3390/vaccines13020204/s1. Supplementary Table S1. Monitoring experimental chickens during the observation period post-challenge.

## References

[1] Alkie T.N., Byrne A.M., Jones M.E., Mollett B.C., Bourque L., Lung O., James J., Yason C., Banyard A.C., Sullivan D. (2023). Recurring Trans-Atlantic Incursion of Clade 2.3. 4.4 b H5N1 Viruses by Long Distance Migratory Birds from Northern Europe to Canada in 2022/2023. Viruses.

[2] Swayne D.E., Suarez D.L., Sims L.D. (2020). Influenza. Diseases of Poultry.

[3] Lee D.H., Bertran K., Kwon J.H., Swayne D.E. (2017). Evolution, global spread, and pathogenicity of highly pathogenic avian influenza H5Nx clade 2.3.4.4. J. Vet. Sci..

[4] Kwon J.H., Bertran K., Lee D.H., Criado M.F., Killmaster L., Pantin-Jackwood M.J., Swayne D.E. (2023). Diverse infectivity, transmissibility, and pathobiology of clade 2.3.4.4 H5Nx highly pathogenic avian influenza viruses in chickens. Emerg. Microbes Infect..

[5] Grund C., Hoffmann D., Ulrich R., Naguib M., Schinköthe J., Hoffmann B., Harder T., Saenger S., Zscheppang K., Tönnies M. (2018). A novel European H5N8 influenza A virus has increased virulence in ducks but low zoonotic potential. Emerg. Microbes Infect..

[6] Runstadler J.A., Puryear W.B. (2024). The virus is out of the barn: The emergence of HPAI as a pathogen of avian and mammalian wildlife around the globe. Am. J. Vet. Res..

[7] Webster R.G., Bean W.J., Gorman O.T., Chambers T.M., Kawaoka Y. (1992). Evolution and ecology of influenza A viruses. Microbiol. Rev..

[8] Wang B., Su Q., Luo J., Li M., Wu Q., Chang H., Du J., Huang C., Ma J., Han S. (2021). Differences in Highly Pathogenic H5N6 Avian Influenza Viral Pathogenicity and Inflammatory Response in Chickens and Ducks. Front. Microbiol..

[9] Kishida N., Sakoda Y., Isoda N., Matsuda K., Eto M., Sunaga Y., Umemura T., Kida H. (2005). Pathogenicity of H5 influenza viruses for ducks. Arch. Virol..

[10] Kim J.-K., Seiler P., Forrest Heather L., Khalenkov Alexey M., Franks J., Kumar M., Karesh William B., Gilbert M., Sodnomdarjaa R., Douangngeun B. (2008). Pathogenicity and Vaccine Efficacy of Different Clades of Asian H5N1 Avian Influenza A Viruses in Domestic Ducks. J. Virol..

[11] Evseev D., Magor K.E. (2019). Innate Immune Responses to Avian Influenza Viruses in Ducks and Chickens. Vet. Sci..

[12] Seekings A.H., Warren C.J., Thomas S.S., Lean F.Z.X., Selden D., Mollett B.C., van Diemen P.M., Banyard A.C., Slomka M.J. (2023). Different Outcomes of Chicken Infection with UK-Origin H5N1-2020 and H5N8-2020 High-Pathogenicity Avian Influenza Viruses (Clade 2.3.4.4b). Viruses.

[13] Kuchipudi S.V., Tellabati M., Sebastian S., Londt B.Z., Jansen C., Vervelde L., Brookes S.M., Brown I.H., Dunham S.P., Chang K.-C. (2014). Highly pathogenic avian influenza virus infection in chickens but not ducks is associated with elevated host immune and pro-inflammatory responses. Vet. Res..

[14] Cornelissen J.B.W.J., Post J., Peeters B., Vervelde L., Rebel J.M.J. (2012). Differential innate responses of chickens and ducks to low-pathogenic avian influenza. Avian Pathol..

[15] Chen J., Liu Z., Li K., Li X., Xu L., Zhang M., Wu Y., Liu T., Wang X., Xie S. (2022). Emergence of novel avian origin H7N9 viruses after introduction of H7-Re3 and rLN79 vaccine strains to China. Transbound. Emerg. Dis..

[16] Maartens L.H., Frizzo da Silva L., Dawson S., Love N., Erasmus B.J. (2023). The efficacy of an inactivated avian influenza H5N1 vaccine against an African strain of HPAI H5N8 (clade 2.3. 4.4 B). Avian Pathol..

[17] Swayne D.E., Kapczynski D.R. (2016). Vaccines and vaccination for avian influenza in poultry. Animal Influenza.

[18] WOAH (2021). Avian influenza (including infection with high pathogenicity avian influenza viruses). Manual of Diagnostic Tests and Vaccines for Terrestrial Animals.

[19] Ali A., Safwat M., Kilany W.H., Nagy A., Shehata A.A., El-Abideen M.A.Z., Dahshan A.-H.M., Arafa A.-S.A. (2019). Combined H5ND inactivated vaccine protects chickens against challenge by different clades of highly pathogenic avian influenza viruses subtype H5 and virulent Newcastle disease virus. Vet. World.

[20] Ibrahim M., Zakaria S., Bazid A.-H.I., Kilany W.H., El-Abideen M.A.Z., Ali A. (2021). A single dose of inactivated oil-emulsion bivalent H5N8/H5N1 vaccine protects chickens against the lethal challenge of both highly pathogenic avian influenza viruses. Comp. Immunol. Microbiol. Infect. Dis..

[21] Zeng X.-Y., Chen X.-H., Wu J.-J., Bao H.-M., Pan S.-X., Liu Y.-J., Deng G.-H., Shi J.-Z., Chen P.-C., Jiang Y.-P. (2020). Protective efficacy of an H5/H7 trivalent inactivated vaccine produced from Re-11, Re-12, and H7-Re2 strains against challenge with different H5 and H7 viruses in chickens. J. Integr. Agric..

[22] Kilany W.H., Safwat M., Mohammed S.M., Salim A., Fasina F.O., Fasanmi O.G., Shalaby A.G., Dauphin G., Hassan M.K., Lubroth J. (2016). Protective efficacy of recombinant turkey herpes virus (rHVT-H5) and inactivated H5N1 vaccines in commercial mulard ducks against the highly pathogenic avian influenza (HPAI) H5N1 clade 2.2.1 virus. PLoS ONE.

[23] Swayne D.E., Spackman E. (2020). Laboratory Methods for Assessing and Licensing Influenza Vaccines for Poultry. Animal Influenza Virus: Methods and Protocols.

[24] Swayne David E., Suarez David L., Spackman E., Jadhao S., Dauphin G., Kim-Torchetti M., McGrane J., Weaver J., Daniels P., Wong F. (2015). Antibody Titer Has Positive Predictive Value for Vaccine Protection against Challenge with Natural Antigenic-Drift Variants of H5N1 High-Pathogenicity Avian Influenza Viruses from Indonesia. J. Virol..

[25] Spackman E., Killian M.L., Spackman E. (2014). Avian Influenza Virus Isolation, Propagation, and Titration in Embryonated Chicken Eggs. Animal Influenza Virus.

[26] EDQM (2021). Monograph, Sterility (2.6.1). European Pharmacopoeia.

[27] EDQM (2021). Monograph, Mycoplasmas (2.6.7). European Pharmacopoeia.

[28] Kilany W., Dauphin G., Selim A., Tripodi A., Samy M., Sobhy H., VonDobschuetz S., Safwat M., Saad M., Erfan A. (2014). Protection conferred by recombinant turkey herpesvirus avian influenza (rHVT-H5) vaccine in the rearing period in two commercial layer chicken breeds in Egypt. Avian Pathol..

[29] Lee E.-K., Song B.-M., Kang H.-M., Woo S.-H., Heo G.-B., Jung S.C., Park Y.H., Lee Y.-J., Kim J.-H. (2016). Experimental infection of SPF and Korean native chickens with highly pathogenic avian influenza virus (H5N8). Poult. Sci..

[30] Reed L.J., Muench H. (1938). A simple method of estimating fifty per cent endpoints. Am. J. Epidemiol..

[31] Percie du Sert N., Hurst V., Ahluwalia A., Alam S., Avey M.T., Baker M., Browne W.J., Clark A., Cuthill I.C., Dirnagl U. (2020). The ARRIVE guidelines 2.0: Updated guidelines for reporting animal research. J. Cereb. Blood Flow Metab..

[32] Duvvuri V.R., Duvvuri B., Cuff W.R., Wu G.E., Wu J. (2009). Role of Positive Selection Pressure on the Evolution of H5N1 Hemagglutinin. Genom. Proteom. Bioinform..

[33] Yeo J.Y., Gan S.K.-E. (2021). Peering into avian influenza a (H5N8) for a framework towards pandemic preparedness. Viruses.

[34] Naguib M.M., Abdelwhab E.M., Harder T.C. (2016). Evolutionary features of influenza A/H5N1 virus populations in Egypt: Poultry and human health implications. Arch. Virol..

[35] Jeong S., Lee D.-H., Kwon J.-H., Kim Y.-J., Lee S.-H., Cho A.Y., Kim T.-H., Park J.-E., Lee S.-I., Song C.-S. (2020). Highly pathogenic avian influenza clade 2.3. 4.4 b subtype H5N8 virus isolated from Mandarin duck in South Korea, 2020. Viruses.

[36] Selim A.A., Erfan A.M., Hagag N., Zanaty A., Samir A.H., Samy M., Abdelhalim A., Arafa A.A., Soliman M.A., Shaheen M. (2017). Highly Pathogenic Avian Influenza Virus (H5N8) Clade 2.3.4.4 Infection in Migratory Birds, Egypt. Emerg. Infect. Dis..

[37] Sultan H.A., Arafa A.-E., Talaat S., Gaballa A.A., Kilany W.H., Elfeil W.K., Shehata A.A., Amarin N. (2019). Efficacy of Clade 2.3.2 H5-Recombinant Baculovirus Vaccine in Protecting Muscovy and Pekin Ducks from Clade 2.3.4.4 H5N8 Highly Pathogenic Avian Influenza Infection. Avian Dis..

[38] Shehata A.A., Sedeik M.E., Elbestawy A.R., Zain El-Abideen M.A., Ibrahim H.H., Kilany W.H., Ali A. (2019). Co-infections, genetic, and antigenic relatedness of avian influenza H5N8 and H5N1 viruses in domestic and wild birds in Egypt. Poult. Sci..

[39] Kandeil A., Hicks J.T., Young S.G., El Taweel A.N., Kayed A.S., Moatasim Y., Kutkat O., Bagato O., McKenzie P.P., Cai Z. (2019). Active surveillance and genetic evolution of avian influenza viruses in Egypt, 2016–2018. Emerg. Microbes Infect..

[40] Cui P., Zeng X., Li X., Li Y., Shi J., Zhao C., Qu Z., Wang Y., Guo J., Gu W. (2022). Genetic and biological characteristics of the globally circulating H5N8 avian influenza viruses and the protective efficacy offered by the poultry vaccine currently used in China. Sci. China Life Sci..

[41] Liu S., Zhuang Q., Wang S., Jiang W., Jin J., Peng C., Hou G., Li J., Yu J., Yu X. (2020). Control of avian influenza in China: Strategies and lessons. Transbound. Emerg. Dis..

[42] Zeng X., Tian G., Shi J., Deng G., Li C., Chen H. (2018). Vaccination of poultry successfully eliminated human infection with H7N9 virus in China. Sci. China Life Sci..

[43] Nielsen S.S., Alvarez J., Bicout D.J., Calistri P., Canali E., Drewe J.A., Garin-Bastuji B., Gonzales Rojas J.L., EFSA Panel on Animal Health and Animal Welfare (AHAW), European Union Reference Laboratory for Avian Influenza (2023). Vaccination of poultry against highly pathogenic avian influenza–part 1. Available vaccines and vaccination strategies. EFSA J..

[44] Luczo J.M., Spackman E. (2024). Epitopes in the HA and NA of H5 and H7 avian influenza viruses that are important for antigenic drift. FEMS Microbiol. Rev..

[45] Cattoli G., Milani A., Temperton N., Zecchin B., Buratin A., Molesti E., Aly M.M., Arafa A., Capua I. (2011). Antigenic drift in H5N1 avian influenza virus in poultry is driven by mutations in major antigenic sites of the hemagglutinin molecule analogous to those for human influenza virus. J. Virol..

[46] Raymond D.D., Stewart S.M., Lee J., Ferdman J., Bajic G., Do K.T., Ernandes M.J., Suphaphiphat P., Settembre E.C., Dormitzer P.R. (2016). Influenza immunization elicits antibodies specific for an egg-adapted vaccine strain. Nat. Med..

[47] Salaheldin A.H., Elbestawy A.R., Abdelkader A.M., Sultan H.A., Ibrahim A.A., Abd El-Hamid H.S., Abdelwhab E.M. (2022). Isolation of genetically diverse H5N8 avian influenza viruses in poultry in Egypt, 2019–2021. Viruses.

[48] Watanabe Y., Ibrahim M.S., Ellakany H.F., Kawashita N., Daidoji T., Takagi T., Yasunaga T., Nakaya T., Ikuta K. (2012). Antigenic analysis of highly pathogenic avian influenza virus H5N1 sublineages co-circulating in Egypt. J. Gen. Virol..

[49] Mo J., Spackman E., Swayne D.E. (2023). Prediction of highly pathogenic avian influenza vaccine efficacy in chickens by comparison of in vitro and in vivo data: A meta-analysis and systematic review. Vaccine.

[50] Spackman E., Suarez D.L., Lee C.-W., Pantin-Jackwood M.J., Lee S.A., Youk S., Ibrahim S. (2023). Efficacy of inactivated and RNA particle vaccines against a North American Clade 2.3.4.4b H5 highly pathogenic avian influenza virus in chickens. Vaccine.

[51] Kumar M., Chu H.-J., Rodenberg J., Krauss S., Webster R.G. (2007). Association of Serologic and Protective Responses of Avian Influenza Vaccines in Chickens. Avian Dis..

[52] Lee D.-H., Kwon J.-H., Noh J.-Y., Park J.-K., Yuk S.-S., Erdene-Ochir T.-O., Lee J.-B., Park S.-Y., Choi I.-S., Lee S.-W. (2016). Pathogenicity of the Korean H5N8 highly pathogenic avian influenza virus in commercial domestic poultry species. Avian Pathol..

[53] Beerens N., Germeraad E.A., Venema S., Verheij E., Pritz-Verschuren S.B.E., Gonzales J.L. (2021). Comparative pathogenicity and environmental transmission of recent highly pathogenic avian influenza H5 viruses. Emerg. Microbes Infect..

[54] Pasick J. (2007). Application of DIVA vaccines and their companion diagnostic tests to foreign animal disease eradication. Anim. Health Res. Rev..

[55] Xu H., Zhu S., Govinden R., Chenia H.Y. (2023). Multiple Vaccines and Strategies for Pandemic Preparedness of Avian Influenza Virus. Viruses.

