# Supplementary material for: Multivalent Inactivated Vaccine Protects Chickens from Distinct Clades of Highly Pathogenic Avian Influenza Subtypes H5N1 and H5N8
Source: Vaccines (Basel). 2025 Feb 19;13(2):204. doi: 10.3390/vaccines13020204 (PMC11860572; doi:10.3390/vaccines13020204)
Supplement: Supplementary file 1 [file vaccines-13-00204-s001.zip › vaccines-3433075-supplementary.pdf]

Supplementary Table S1. Monitoring experimental chickens during the observation period post-challenge

**Group(s)**[*] (*n*=15 for each group)	**Obs.**[†]	**Days-post-challenge (DPC)**										**Protection**[‡] **%**	**Mortality %**
		**1**	**2**	**3**	**4**	**5**	**6**	**7**	**8**	**9**	**10**		
GA1: VC-2.2.1.2	N	13	12	13	14	15	15	15	15	15	15	15/15 (100%)	0/15 (0%)
	S	2	3	2	1	0	0	0	0	0	0		
	D	0	0	0	0	0	0	0	0	0	0		
GA2: VC-2.3.4.4b, CO	N	13	12	13	14	15	15	15	15	15	15	15/15 (100%)	0/15 (0%)
	S	2	3	2	1	0	0	0	0	0	0		
	D	0	0	0	0	0	0	0	0	0	0		
GA3: VC-2.3.4.4b, DO	N	14	14	13	14	15	15	15	15	15	15	15/15 (100%)	0/15 (0%)
	S	1	1	2	1	0	0	0	0	0	0		
	D	0	0	0	0	0	0	0	0	0	0		
GB1: UC-2.2.1.2	N	0	0	0	-[§]	-	-	-	-	-	-	N/A[**]	15/15 (100%)
	S	15	12	0	-	-	-	-	-	-	-		
	D	0	3	12	-	-	-	-	-	-	-		
GB2: UC-2.3.4.4b, CO	N	0	0	0	0	-	-	-	-	-	-	N/A	15/15 (100%)
	S	15	10	5	0	-	-	-	-	-	-		
	D	0	5	5	5	-	-	-	-	-	-		
GB3: UC-2.3.4.4b, DO	N	7	2	0	0	0	0	-	-	-	-	N/A	15/15 (100%)
	S	8	13	13	9	3	0	-	-	-	-		
	D	0	0	2	4	6	3	-	-	-	-		
GB4: NC	N	15	15	15	15	15	15	15	15	15	15	N/A	0/15 **(0%)**
	S	0	0	0	0	0	0	0	0	0	0		
	D	0	0	0	0	0	0	0	0	0	0		

[*] *VC: vaccinated challenged, UC: unvaccinated challenged, NC: negative control, CO: chicken origin, DO: duck origin.*

[†] *N: normal, S: Sick, D: Dead.*

[‡] *Protection (against mortality) %: percentage of survivors in the group out of the initial number of birds in the group.*

[§] *Hyphen symbol indicates no observation is recorded given that all the birds were already dead.*

[**] *Not applicable.*
